# Implications of summer breeding phenology on demography of monarch butterflies

**DOI:** 10.1111/1365-2656.70004

**Published:** 2025-02-17

**Authors:** Diane M. Debinski, Norah Warchola, Sonia Altizer, Elizabeth E. Crone

**Affiliations:** ^1^ Ecology Department Montana State University Bozeman Montana USA; ^2^ Ecology and Evolutionary Biology Iowa State University Ames Iowa USA; ^3^ Odum School of Ecology University of Georgia Athens Georgia USA; ^4^ Department of Biology Tufts University Medford Massachusetts USA; ^5^ Present address: Department of Evolution and Ecology University of California Davis Davis California USA

**Keywords:** *Asclepias*, asynchrony, climate change, *Danaus plexippus*, migration, milkweed, phenology

## Abstract

1. Phenological changes have been widely documented in animal and plant responses to directional environmental change. However, predicting the consequences of these shifts for species interactions and population viability requires knowledge of vital rate responses to biotic and abiotic drivers.
2. Here, we paired long‐term phenology data documenting monarch butterfly abundance and occurrence of their milkweed hostplant with outdoor experiments in the central United States to ask how changes in spring arrival times to monarch breeding sites affect their development, survival, and within‐season population growth.
3. Monarch arrival times did not change across the 17 years of monitoring, but the peak abundance of monarchs, which occurred just prior to their fall migration, shifted 9 days later in 2019 as compared to 2003. Summer population growth declined from 2003 to 2019, significant in ~80% bootstrap calculations. Phenological changes in milkweed occurrence mirrored changes in monarch abundance, happening later through time. Our field experiment showed that early season larval survival was highest when the timing of hatching matched the average timing of the first natural monarch cohort; survival was lowest when egg hatching shifted 14 days earlier.
4. The results of our study indicate that earlier arrival of adult monarchs to summer breeding habitat would be costly for monarchs—but field survey data show that arrival times have not changed to date. Instead, the local changes we observed in the timing of peak abundance occurred towards the end of the breeding season, not the onset. At present, we conclude that changes in early season phenology are not a threat to eastern North American monarchs living in the central United States, but drivers of breeding‐season growth rates and changes in late‐season phenology merit further study, both in the central United States and in other parts of the monarch's range.

## Graphical abstract

This research showed that changes in early season phenology are not a threat to eastern North American monarchs living in the central United States, but drivers of breeding‐season growth rates and changes in late‐season phenology merit further study, both in the central United States and in other parts of the monarch's range. Photograph by Cody Prouty.

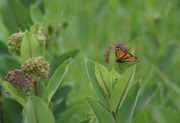

## 1 INTRODUCTION

Ecologists widely recognize that the timing of life cycle events for many species is shifting in response to directional environmental change (Cohen et al., 2018). This change is especially evident for migratory species that move long distances seasonally between breeding and wintering grounds (Gill et al., 2019; Lawrence et al., 2022). Migratory patterns are most well‐studied in birds, and earlier arrival of migrants on breeding grounds has been reported to align with temperature and climate‐mediated changes in spring vegetation (Koleček et al., 2020; Youngflesh et al., 2021). However, other studies show increasing phenological asynchrony between spring green‐up and the arrival of migrants (Mayor et al., 2017; Tomotani et al., 2018). Importantly, a multi‐decadal synthesis of European bird data showed that species with declining populations had not advanced the timing of their spring migration, whereas species with stable or increasing populations had advanced the timing of their migration (Møller et al., 2008). It is not clear whether the shifts observed in avian populations occur more broadly across other groups of migratory species, and whether such changes benefit or reduce population viability.

The monarch butterfly is an iconic migratory insect species, and changes in its summer breeding phenology could significantly affect its population dynamics. The eastern North American population of monarch butterflies (*Danaus plexippus*) extends broadly across much of the central and eastern portions of the United States and the southern part of Canada. This population undergoes an annual long distance fall migration to wintering grounds in Mexico (Brower et al., 2006). After spending up to 5 months at their wintering sites, monarchs recolonize their breeding range, moving northward via multiple reproductive generations (Flockhart et al., 2013; Pleasants et al., 2015; Zipkin et al., 2012). The phenology of monarch spring arrival on breeding grounds depends on a variety of factors, including temperature and photoperiod shifts that trigger northward movement, circadian clocks in their antennae that influence directional flight, a magnetic compass, and spring weather conditions (Guerra & Reppert, 2015; Howard & Davis, 2015). Initiation of the return of monarchs northwards likely co‐evolved with spring milkweed host plant emergence, so that butterflies can oviposit on fresh host plants (Guerra & Reppert, 2015). Monarch larvae feed only on milkweeds (in the family Apocynaceae) (Malcolm & Brower, 1989) and the particular milkweed species used varies by region (Pocius et al., 2018). Because of the length of their migration and their dependence on milkweed resources, monarchs may be particularly vulnerable to phenological mismatches. The degree of monarch‐milkweed synchrony during arrival to and departure from their breeding grounds has the potential to shape monarch population dynamics (Crewe et al., 2019; Yang & Cenzer, 2020). Synchrony with non‐milkweed nectar plants may also be important, but because most adult butterflies are opportunistic in their use of nectar plants, we focus here on the milkweed and monarch synchrony.

Monarchs have experienced population declines in recent decades, as evidenced by analysis of long‐term trends in overwintering abundance (Brower et al., 2012; Semmens et al., 2016). Several factors have been implicated in these declines, including the loss of overwintering habitat, herbicide‐related and landscape management‐related declines in native milkweed, loss of nectar resources and stopover sites for migration, planting of non‐native tropical milkweed which encourages winter breeding, and disease‐induced mortality (Majewska et al., 2022; Pleasants & Oberhauser, 2013; Satterfield et al., 2018; Vidal & Rendón‐Salinas, 2014). Changes in phenology could contribute to, or help mitigate, these declines. For the eastern monarch population, both the timing of the fall migration southward (Culbertson et al., 2022), and spring arrival northward have gotten later over recent decades (Howard & Davis, 2015), although some portions of the eastern population have shown no change in fall migration timing (Ethier & Mitchell, 2023). Declines in the eastern North American monarch population (Brower et al., 2012; Pelton et al., 2019; Thogmartin et al., 2017; Vidal & Rendón‐Salinas, 2014) have led to conservation efforts from national (USFWS, 2024) and international (Normile, 2023) perspectives.

Milkweed‐monarch phenology can affect monarch survival and reproduction in several ways. Monarch larval performance depends on seasonal changes in host plant quality (Yang et al., 2020). Larvae prefer younger plant tissues and regenerating stems (Bergström et al., 1994; Haan & Landis, 2019b). Typically, plant nutrient levels (e.g. nitrogen) decrease while physical and chemical defence levels (e.g. cardiac glycosides, tannins) increase over the growing season (Barton & Koricheva, 2010; Schroeder, 1986). In addition, early season milkweeds or newly regenerated stems may harbour fewer natural enemies of young larvae and fewer competitors (Pocius et al., 2022). Shifts in the timing of the monarch–milkweed interaction could arise from changes in either species. Flowering time of common milkweed (*Asclepias syriaca*) depends on temperature (3.93 days/°C) and there is some evidence that start and end of blooming time is advancing (Howard, 2018). As such, we might expect earlier arrival of monarchs to benefit their fitness if milkweed phenology is advancing.

There has been limited assessment of asynchrony between monarchs and their milkweed host plants under natural conditions or via field experiments (but see Yang & Cenzer, 2020) and how such changes could affect monarch population dynamics. Here, we combined observational, experimental, and quantitative approaches to ask: (1) Is the phenology of monarch butterflies and/or milkweed in the central United States changing through time? and (2) If the phenology of monarch butterflies changed, how might this affect larval development and survival? Our study included three components: First, we quantified shifts in abundance and phenology of adult butterflies to assess whether monarch arrival, departure and peak abundance times changed over a 17‐year period. Second, we conducted manipulative field experiments to measure the impact on larval survival of eggs laid before, during or after reproductively active monarchs moved into the area. If monarchs and milkweeds become phenologically mismatched, we would expect to see a decrease in monarch survival. Third, to aid in the interpretation of monarch phenology trends, we compiled historical specimen data for the most abundant milkweed species at our field site, *A. syriaca*, and analysed trends in specimen collection dates through time, as a coarse estimate of milkweed phenology. Combining the field experiment with long‐term patterns of phenological change in both monarchs and milkweed helps contextualize the potential effects of phenological mismatches in this study system.

## 2 MATERIALS AND METHODS

### 2.1 Study system

We conducted monarch monitoring and a field experiment in the central part of the United States (Camp Dodge headquarters of the National Guard in Iowa) which is in the core of the eastern monarch population's summer breeding range. Camp Dodge is a 1740 ha military installation and includes large areas of restored prairie habitat (20–40 ha), small prairie remnants (4–8 ha in size), wooded areas, and large areas of turf grass. Transect surveys were conducted in closely cropped turf grass with nectar sources that are mostly weeds such as dandelions (*Taraxacum* sp.), clovers (*Trifolium* spp.) and asters (Asteraceae). The transect locations are not prime butterfly habitat, but are located within the matrix of a relatively large grassland (100's of ha) that supports large populations of common milkweed (*A. syriaca*) and swamp milkweed (*A. incarnata*). Monarchs are large butterflies, highly mobile and move freely through the landscape. They were seen regularly on transects. The Camp Dodge property thus contributes substantially to the area of grassland habitat for monarch butterflies within the highly fragmented central US agricultural landscape.

### 2.2 Transect surveys of adult monarchs

Butterfly surveys were conducted at mid‐day by a single observer (Harlan Ratcliff) from Monday to Friday each week and from April to November during 2003–2019. Whereas these surveys started somewhat less formally in 2003, by 2007 the surveys were conducted using a Pollard‐transect‐like survey (Pollard, 1977). Initially the transect length was 3.2 km, although from late 2015–2019, the transect length was 2.4 km. Surveys were conducted from ~1130 to 1230 h and each walk was ~45 min in duration.

We extracted monarch abundance data by date from the complete list of butterfly species observed. We evaluated trends in monarch abundance and phenology by fitting additive generalized models (GAMs) to abundance as a function of day of year (DOY) and year (Diamond et al., 2014; Hodgson et al., 2011). See also Stemkovski et al. (2020) for a similar analysis of bee populations. GAMs were fit using the default settings in the R package mgcv (Wood, 2011), and included an offset of log(route length), that is 3.2 km from 2003 to 2014 and 2.4 km from 2015 to 2019, a statistical technique that converts the total count to the count per distance (see Zuur et al., 2009, their chapter 9, pp. 240–241). This allowed us to standardize the counts of butterflies per distance walked. The full model included effects of year, day of year and their interaction, that is an s(DOY, year) term in the GAM syntax. The interaction term was included to test whether the effects of temperature would differ among years. We evaluated the statistical significance of these effects using marginal hypothesis tests, implemented manually by fitting appropriate reduced models (see Section 3) and comparing them with likelihood ratio tests. We also assessed cumulative growing degree days (cGDD) as a predictor of phenology. Because cGDD was a worse predictor of abundance and phenology than DOY (Table S1; Figure S1), these results are not discussed further.

We used the fitted GAM to describe changes in monarch abundance and phenology using: (1) relative abundance, calculated as the area under the GAM curve for each year (cf. Wepprich et al., 2019), (2) the date of peak activity of the first monarch breeding generation, defined as the date of the first peak (local maximum) of the fitted GAM, (3) the date of peak activity of the last monarch breeding generation, defined as the date of the next‐to‐last peak (local maximum) of the fitted GAM, and (4) the date of peak activity of the combined last monarch breeding generation and fall migrants moving south into Iowa, defined as the date of the last peak (local maximum) of the fitted GAM. To estimate changes in abundance, we used the ratio of the next‐to‐last to first peak height, as a rough metric of change in abundance during the summer breeding season (“summer growth”), and the ratio of the last to first peak height, as a rough metric of “total” growth during the summer breeding season.

We used simple linear models to test for linear trends in each metric through time (across years). Note that the overall GAM analysis provides test statistics for whether abundance and phenology are changing over time, but not whether these changes are consistent with monotonic increases or decreases. Therefore, we used bootstrapping (James et al., 2021) to assess statistical confidence in these trends. Specifically, we resampled the original data with replacement, re‐fit the winning model to the bootstrap sample, and then calculated linear trends in the derived metrics from the model fit to the bootstrap sample. We recorded the proportion of models fit to bootstrapped data that showed significant trends in the same direction as the original model. This analysis included 5000 bootstrapped datasets.

### 2.3 Field experiment of larval survival

To assess how a shift in monarch spring arrival times might affect larval development and survival, we conducted a field experiment during May–July, 2020. We included three levels of phenological overlap between larvae and host plants in terms of the date that eggs hatch and caterpillars begin feeding: (1) current dates of first larval presence (described below), (2) 2 weeks earlier than current dates and (3) 2 weeks later than current dates. For simplicity, we refer to these levels of phenological overlap as current, earlier and later, respectively.

The field experiment was initiated with eggs obtained from the progeny of wild monarchs captured during their annual fall migration October 2019 in Athens, GA and St Marks, FL. Adults were overwintered in Georgia in Percival incubators set to 12°C (daily high on the overwintering grounds) with photoperiod set weekly to mimic sunrise and sunset at the eastern migratory monarchs' natural wintering sites near Angangueo, Mexico. In early April 2020, these same adult monarchs were mated in 0.6 m^3^ mesh cages held at 28°C and fed ad‐libitum with a 20% honey water solution. The progeny of 10 wild females were examined for the presence of *Ophryocystis elektroscirrha* following Altizer et al. (2000), and only uninfected butterflies were used for subsequent mating and oviposition. In May 2020, we established eight non‐inbred genetic lineages, and for each lineage, mated females oviposited onto potted *A. syriaca* plants. Eggs were laid in three cohorts separated by 14 days, with egg collection on May 4–5, May 18–19, and June 1–2. Approximately 50 eggs per lineage (400 eggs in total per time interval) were obtained for each cohort. The same individual females were used to obtain eggs for each cohort, and females were held in glassine envelopes at 22°C and fed every second day between egg collection. Eggs on stalk cuttings were shipped overnight to Camp Dodge, Iowa, within 2 days of oviposition, and larvae remained on natal stalks indoors at 22°C until 1 day post‐hatching.

**FIGURE 1:**
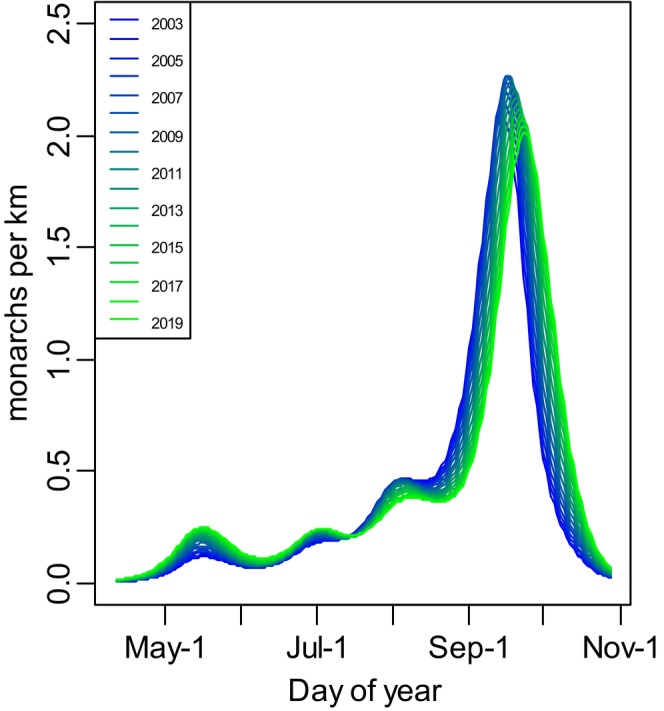
Smoothed relationship between day of year and adult monarch count, based on the winning generalized additive model with an interaction between year and day of year (GAMs).

Target oviposition dates were selected following analysis of field data on monarch surveys (as described in the previous section, and see Section 3). GAMs indicated that the first adult monarchs were seen, on average, in mid‐May each year (Figure 1), with consistency in arrival times among years. We assumed that females in the field would lay eggs at the site immediately upon arrival. Eggs take 3–4 days to hatch (Haan & Landis, 2019a). Thus, larvae were placed on plants in the field on May 13 (earlier), May 25–26 (current) and June 8 (later) (see Table S2 for details). Two old field sites, approximately 3 km apart, with abundant milkweed (hundreds of *A. syriaca*, common milkweed) were used for this field experiment. Within each site, we used a random number generator to select a cardinal direction to walk from near the centre of the patch. The first milkweed encountered that was 30 cm in height or taller was selected for larval placement. We used this method to select the next plants, walking a minimum of 2 m from the previous milkweed plant until there were seven plants selected at each site. The 2 m distance was used so that one cage would never shade another, limiting potential effects of abiotic stress.

Larvae were placed on caged plants to allow for semi‐natural levels of insect predation and parasitism. Before placing the larvae on the plant, we looked for and removed any monarch larvae or eggs; other insects and spiders present were left undisturbed so that we could assume that any parasitism that occurred was a result of the local insect community. Each focal milkweed plant was enclosed in a 1.22 m tall steel wire cage (5.08 × 10.16 cm grid) with a 35.6 cm diameter. The steel wire cage was covered with a nylon mesh 2 m tall (5 mm hexagonal mesh) that was knotted at the top and formed a skirt at bottom. Tent stakes were used to hold the mesh firmly against the soil. We placed 30 larvae on each plant during trials 1 and 2 (a total of 420 larvae), and 20 larvae/plant in trial 3 (a total of 280 larvae). For trial 3, females laid fewer eggs than expected, thus limiting the total sample sizes. We used 30 larvae per plant because monarch survival probability from egg to pupation in the field is low [often less than 0.05 (Grant et al., 2020)], with mortality especially high among the early instar larval stages (Nail et al., 2015). We monitored monarch survival every 2–3 days until they eclosed as adults. In the few cases where defoliation occurred, we moved an adjacent milkweed stalk into the cage to prevent larval starvation. Milkweed was examined carefully immediately before and after moving caterpillars to ensure that counts remained the same.

We analysed survival using binomial family, logit link, generalized linear mixed models (GLMMs) with the number of larvae released in each cage as the number of trials and the number of adults eclosing as the number of events. Models included fixed effects of release timing (current, earlier and later dates) and random effects of cage identity. We also analysed development time of successful adults (i.e. time between release as small larvae in the field and eclosion) using gaussian family, identity link GLMMs, with fixed effects of release timing and random effects of cage identity. In both cases, models were fit using the lme4 package (Bates et al., 2015) and statistical significance was evaluated using type II marginal hypothesis tests implemented with the car::Anova() function (Fox & Weisberg, 2019) in R version 4.0.2 (R Core Development Team, 2020).

### 2.4 Milkweed herbarium data

We compiled herbarium records for *A. syriaca* in the three adjacent Midwestern (i.e. central) US states with similar weather patterns, vegetation, geology and land‐use near the study site (Minnesota, Wisconsin and Iowa) from 1970 (shortly before the recent period of rapid climate change; cf. Bartomeus et al., 2011; Dorian et al., 2023) through 2018. We included three states because very few specimens (*N* = 15) had been collected from Iowa, compared to Minnesota (*N* = 100) and Wisconsin (*N* = 348). We gathered herbarium records primarily from online sources (Consortium of Midwest Herbaria, https://midwestherbaria.org/portal/) and a visit to the Iowa State University Herbarium. Our approach is similar to that of Crone et al. (2024) who used a combination of historical herbarium data and more recent field‐based data to estimate phenological overlap between checkerspot butterflies and multiple nectar plant species across several decades. They found that historical data had some power in predicting patterns of phenology in more recent time periods. They did, however, note that models of phenological activity patterns based on herbarium specimen data represent a larger geographic area, and thus would be expected to represent a greater range of conditions than a particular field site.

We analysed phenology using quantile regression, implemented with the “quantreg” package in R (Koenker et al., 2018). Following Michielini et al. (2021), we analysed trends in the collection date (DOY) in relation to the year of collection, and used the 0.1, 0.5 and 0.9 quantiles to represent the onset, median and end of the above‐ground activity period of milkweed. We also reviewed the database of 56,675 nectar and host plant records from the three states to determine whether there were seasonal differences in collection during two periods of time, 1970–1990 (historic data) and 1998–2018 (recent data), that would create a bias in our results. We found no bias; the percent of the samples that comprised recent relative to historic dates were virtually the same during each of the three seasonal periods (data not shown). The vast majority of herbarium specimens collected are reproductive plants. For example, 97% of the herbarium specimens of *Chelone glabra* analysed in a phenological analysis were in flower (Crone et al., 2024), so these dates should be interpreted as a measure of relative phenology, not the absolute length of the period when *A. syriaca* was growing and producing above‐ground vegetation.

## 3 RESULTS

### 3.1 Transect surveys of adult monarchs

We evaluated the transect survey data of adult monarch butterflies to determine whether their phenology was changing over time. Monarch butterfly abundance generally increased from early to late in the breeding season, as evidenced by a significant main effect of DOY (comparison of the model with additive effects of year and DOY to one with year only: *χ* ^2^ = 207.8, edf = 0.36, *p* < 0.001). This main effect reflected the expected growth of the monarch butterfly population through the breeding season (Figure 1). Monarch butterfly abundance did not change in a simple directional way between years (comparison of the model with additive effects of year and DOY to one with DOY only: *χ* ^2^ = 0.14, edf = 1.02, *p* = 0.706). However, there was an interaction between DOY and year, indicating a change in abundance and phenology through time (comparison of the model with additive effects of year and DOY to one with their interaction: *χ* ^2^ = 24.39, edf = 8.56, *p* = 0.004; see Figure 1).

**FIGURE 2:**
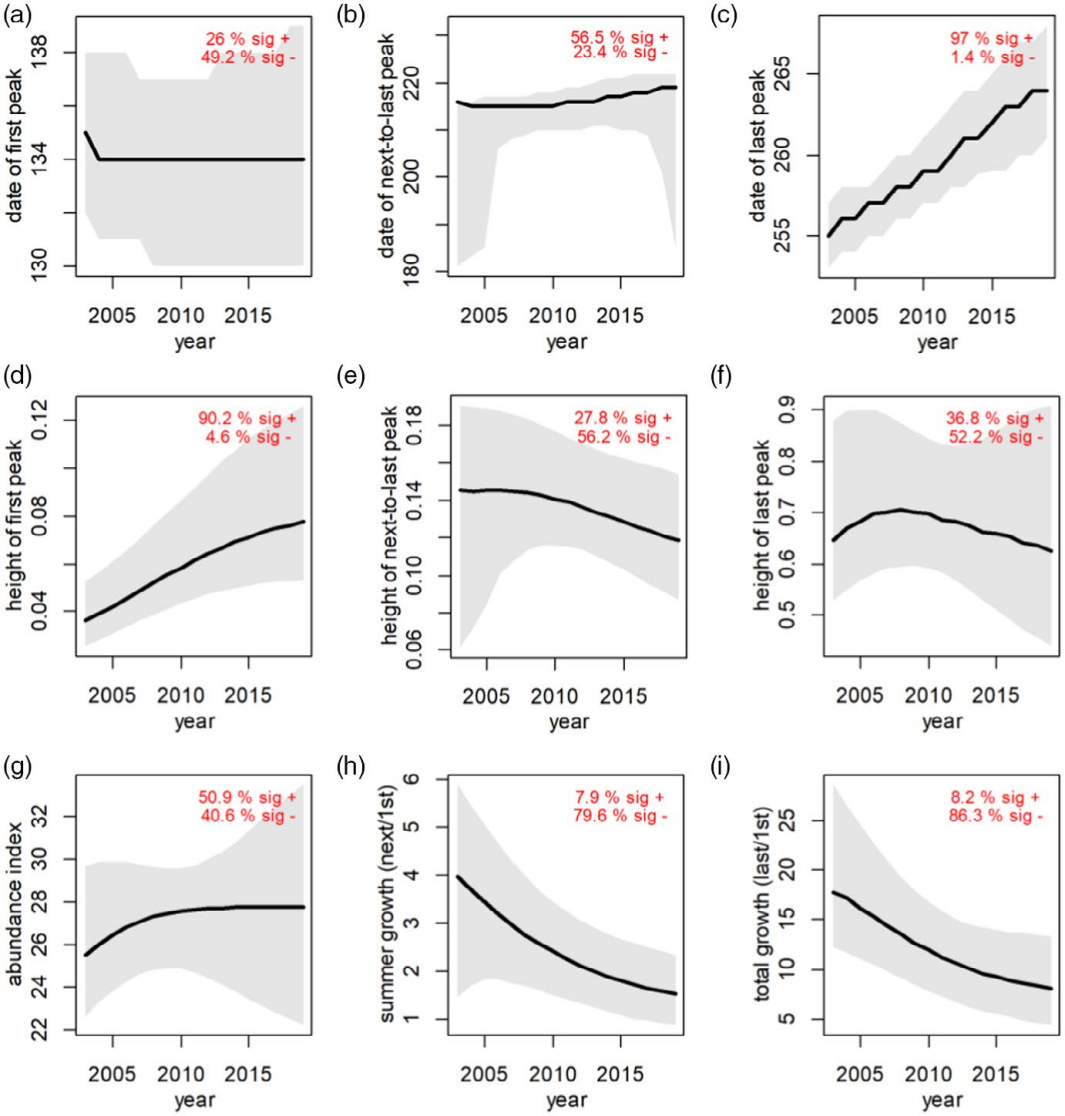
Metrics derived from fitted GAMs for monarchs at Camp Dodge IA, including day of year of first, next‐to‐last and last peaks, respectively, using Julian dates (a–c); height of first, next‐to‐last and last peaks, as a rough metric of relative abundance (d–f); and abundance index, calculated as the area under the fitted GAM curve, ratio of the next‐to‐last to first peak height, as a rough metric of change in abundance during the summer breeding season, and ratio of the last to first peak height, as a rough metric of “total” growth during the summer breeding season (g–i). Black line shows estimates extracted from GAM fitted to full data set, the grey envelope shows 16% to 85% envelope of models fit to bootstrapped data sets (analogous to one standard error), and the red numbers indicate the proportion of bootstrapped data sets with significant (*p* < 0.05) positive (+) and negative (−) associations. GAM, generalized additive model.

To understand the implications of the year × DOY interaction, we explored predictions of the fitted model (Figure 2a–i). Of particular interest to our experiment, arrival timing was remarkably consistent through time (Figure 2a). However, the date of the last peak, which occurred just prior to fall migration, was 9 days later in 2019 as compared to 2003 (Figure 2c, significant in 95% of bootstrap simulations). The peak number of monarchs in the early season increased from about 0.04 to 0.08 monarchs per survey from 2003 to 2019 (Figure 2d). Summer growth went from 4.0 in 2003 to <2.0 in 2019, a decline of over 50% (Figure 2h), and ‘total’ growth went from 17.7 in 2003 to 8.1 in 2019, a decline of 50% (Figure 2i). These abundance and growth trends were not significant in 95% of bootstrap simulations, but they suggest that even though more monarchs were arriving to Camp Dodge over time, the same number (or slightly fewer) were leaving.

### 3.2 Field experiment of larval survival

**FIGURE 3:**
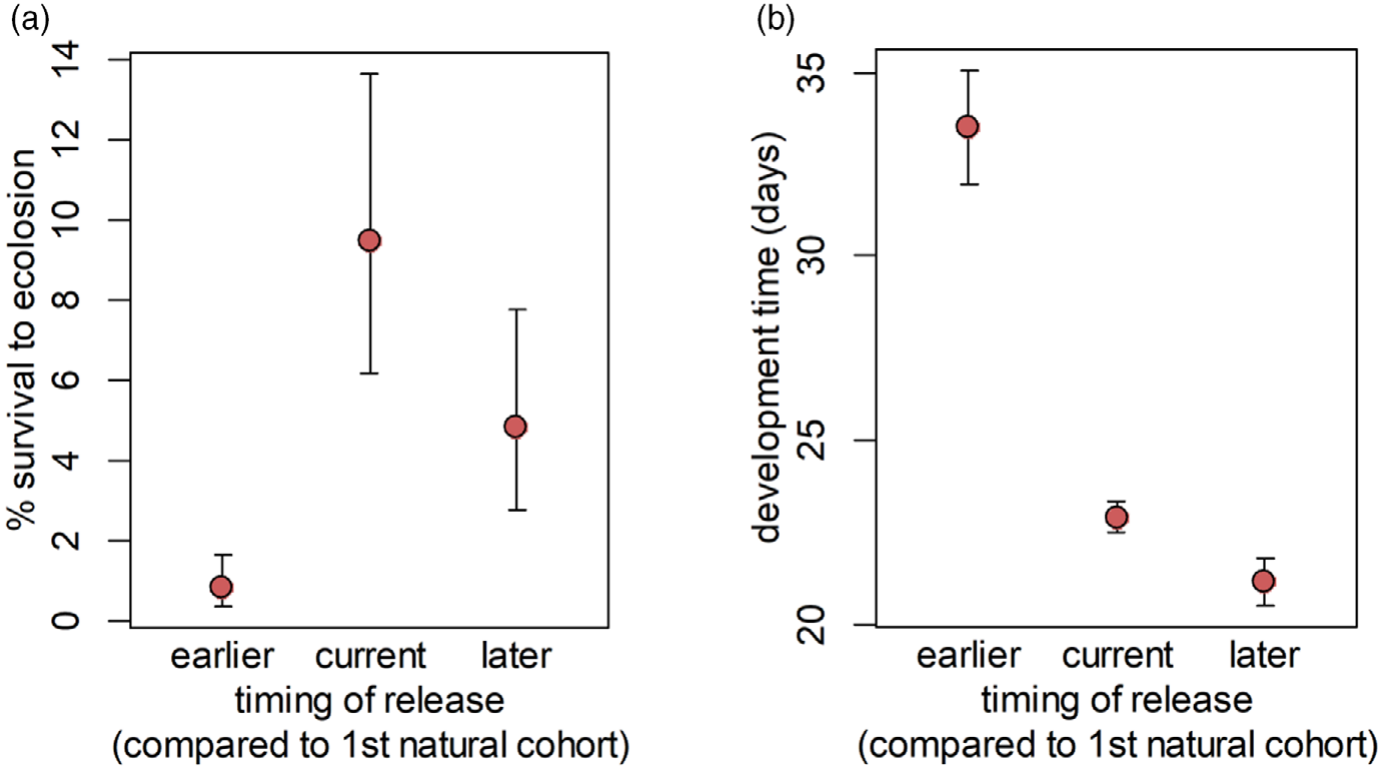
Demographic fate of monarch larvae as a function of release date measured via (a) survival to eclosion, and (b) development time (days from release to eclosion) of butterflies that survived. Sampling units are total individual larvae across all plants and sites. Error bars show 83.4% confidence intervals, which makes non‐overlapping error bars equivalent to a significant pairwise comparison (Payton et al., 2003).

The field experiment of larval survival allowed us to determine whether a shift in larval phenology would affect survival. Larval survival to eclosion differed significantly among release dates (*χ* ^2^ = 17.1, df = 2, *p* < 0.001; Figure 3a). Survival was highest when timing of release matched the average time of first natural cohort (9.5%, 95% CI 5.0%–15.8%). Survival was lower (4.8%, 95% CI 2.1%–9.3%), but not significantly different when released later than the first natural cohort. Notably, survival was lowest (0.8%, 95% CI 0.2%–2.1%) when released earlier than the first natural cohort. This difference in survival could be due in part to slower development of the first cohort of larvae (Figure 3b). Development time was about 10 days longer in the earlier cohort (mean = 33.5 days, 95% CI 31.2–35.7) than in the current (mean = 22.9 days, 95% CI 22.3–23.5 days) or later (mean = 21.1 days, 95% CI 20.2–22.1 days) cohorts (*χ* ^2^ = 96.9, df = 2, *p* > 0.001).

### 3.3 Milkweed specimen data

**FIGURE 4:**
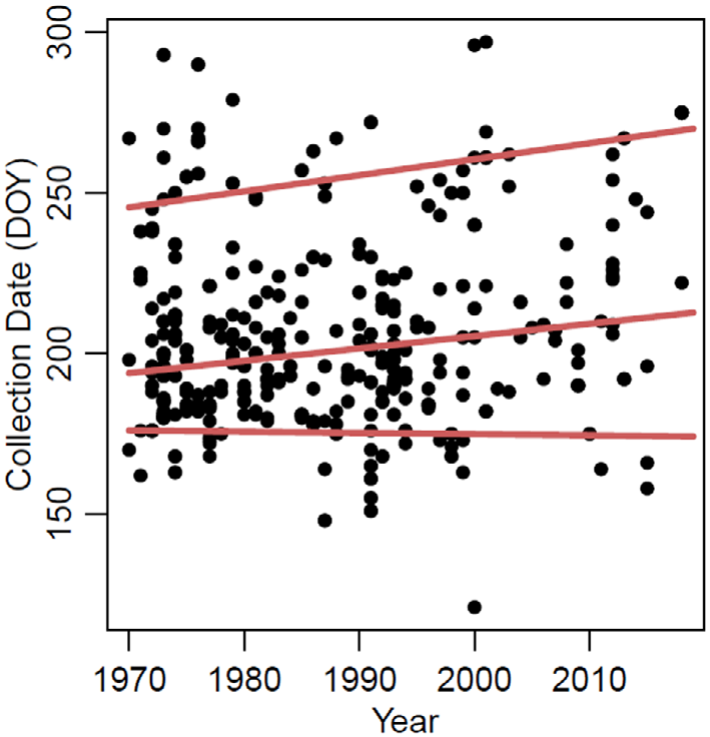
Changes in milkweed (*Asclepias syriaca*) phenology through time, as inferred from herbarium specimens collected in the states of Iowa, Wisconsin and Minnesota. Lines indicate trends in the end (0.9 quantile, top line), median (0.5 quantile, middle line) and onset of collection dates (0.1 quantile, bottom line.)

We evaluated the herbarium data to determine whether milkweed phenology was changing over time. Historical specimens showed no evidence of phenological mismatch between monarch butterflies and *A. syriaca* in our region. The onset of flowering (inferred from the 0.1 quantile of collection dates) remained similar through time (slope ± SE = −0.038 ± 0.127 days/year, *t* = −0.30, *p* = 0.762, *N* = 463 specimens; Figure 4). The median (0.5 quantile) and end (0.9 quantile) of collection dates delayed through time (slope ± SE = 0.385 ± 0.154 days/year, *t* = 2.50, *p* = 0.013 and slope ± SE = 0.500 ± 0.213 days/year, *t* = 2.35, *p* = 0.019, respectively; Figure 4). Notably, the lengthening of flowering activity (0.500 days/year) was similar to the change in monarch late peak abundance dates (9 days later over 16 years, i.e. 0.56 days/year).

## 4 DISCUSSION

The results of our observational and experimental findings are broadly consistent, indicating that phenological shifts in arrival times would be costly to monarch fitness, and have not occurred to date. Our results contrast with volunteer‐based reports showing first sightings of monarch butterflies in North America have gotten later over recent decades (Howard & Davis, 2009). However, changes in first sighting dates may be driven by declines in abundance, not changes in phenology (Edwards & Crone, 2021). Curve‐fitting methods like the GAMs used in our analysis are well‐established for estimating changes in phenology and abundance from repeated transect surveys (Edwards et al., 2024). In addition, first sighting dates can be affected by multiple variables including detectability, sampling effort, and population size (Inouye et al., 2019; Miller‐Rushing et al., 2008; van Strien et al., 2008). The value of the type of monarch data presented here is that it was all collected by one person with incredible regularity over the course of 17 years, thus avoiding some of the issues of lack of precision and continuity associated with citizen science data (Howard, 2018).

The lack of change in the onset of breeding found in our study contrasts with many butterfly species, which tend to be advancing in the onset of flight activity through time (Altermatt, 2012; Kharouba & Vellend, 2015; Macgregor et al., 2019; Michielini et al., 2021; Parmesan & Yohe, 2003), though there are exceptions to this pattern (Colom et al., 2022; Hällfors et al., 2021). Yet, multi‐species studies on phenological shifts in butterflies using observational data (as cited here) often exclude migratory species, because their phenological onset events are likely influenced not by local climatic conditions but by broader climatic patterns across larger geographic areas. The lack of change in monarch butterfly phenology that we observed is also consistent with some species of migratory birds (Mayor et al., 2017), even though the arrival time of most bird species is advancing and birds are some of the best examples of phenological mismatches of consumers with their food sources (Kharouba & Wolkovich, 2023).

Although we did not observe a phenological mismatch between monarch butterflies and their host plants, phenology was an important predictor of survival and development, and the largest effect was lower survival and longer development in the ‘earlier’ arrival treatment. Our results complement research in the western United States by Yang et al. (2020) and Yang and Cenzer (2020), where developmental success of larval monarchs showed a strong phenological signal and early season monarch survival was limited by the biomass of young milkweed plants. Host plant limitation is probably less of an issue in the central United States (pers. obs.) and eastern (Gilmour & Kharouba, 2022) portion of the monarch range. In our study, it seems more likely that cool temperatures limited early season survival by slowing monarch growth rates and affecting developmental processes. Monarch larval development is temperature‐dependent (Oberhauser & Solensky, 2004) and previous studies conducted at constant temperatures suggest that larvae develop best at 27°C with an upper lethal thermal limit of 42°C and a lower developmental thermal limit of 11–12°C (Nail et al., 2015; Zalucki, 1982). Kharouba and Yang (2021) found a direct, positive effect of warming from 26 to 30°C on monarch larval growth rate, increasing it by 27%. Similarly, Zalucki (1982) showed that monarch survival increased in the laboratory (in the absence of natural enemies and with ample food) between 15 and 29°C. At Camp Dodge, the average maximum temperature during our experiment was 15.8°C for the “earlier” treatment (May 13–20), 20.8°C for the ‘current’ treatment (May 25–May 31), and 24.1°C for the “later” treatment (June 8–15; data from https://www.timeanddate.com), suggesting generally better conditions for development in early June relative to mid‐May. Thus, even without an actual phenological shift, variations in monarch arrival time among years could have implications for larval survival.

Larval survival across all three treatments was relatively low (0.8%–9.5%), but this is not surprising. Butterfly larvae are subject to high predation and parasitism rates (Feeny et al., 1985). Black swallowtail (*Papilio polyxenes*) egg to fourth instar larval survival was estimated at 2%–6% (Feeny et al., 1985) and monarch egg to adult survival in other studies was estimated at 1.4% (Grant et al., 2020) and 4.3% (Nail et al., 2015). Longer development time, as in our earlier treatment, leaves the larvae subject to predation and parasitism for an extended period, which could have implications on survival.

In contrast to no change in phenology of monarch arrival and milkweed onset, both date of last peak of monarch abundance (Figure 2c) and the end of milkweed collection dates (Figure 4) extended later into the fall over time. The change in timing of both activities was similar (ending 0.56 days/year later for monarchs and 0.50 days/year later for milkweeds), suggesting phenological change but not phenological mismatch. Past studies have shown that plants with later flowering seasons, like *A. syriaca*, are likely to extend flowering later into the fall (Aldridge et al., 2011; Sherry et al., 2007). The shift in timing in the date of the last peak for monarch abundance at Camp Dodge, which includes the fall migrants coming south into Iowa, is quantitatively similar to observations of fall migrating monarchs from Cape May, New Jersey, United States (Culbertson et al., 2022), which have been getting later (0.57 days/year; but see Ethier and Mitchell (2023) for no change in the timing of fall migration in Ontario, Canada). Culbertson et al. (2022) posit that longer growing seasons, northward expansion of milkweed ranges, and potentially a northward expansion of the monarch range may be driving later migration in monarchs. Our results suggest that a longer milkweed flowering season could be one factor contributing to delays in monarch departure. Migration delays have been observed in some bird species, particularly shorter‐distance migrants (Haest et al., 2019), and it is interesting to speculate that monarchs are responding to extended periods of resource availability. Extended time of resource availability might benefit monarchs by providing prolonged resources, but the trade‐offs include increased exposure to late‐season predators or parasitoids.

Compared to the onset of the growing season, events at the end of the growing season have been termed ‘the forgotten season’ and are generally less well studied (Ekholm et al., 2019), as is evidenced by the fact that our study was designed to test effects of spring arrival but not fall departure. And while we found that both monarchs and milkweeds are changing in concert with later phenological activities in more recent time, it is possible that these changes could lead to mismatches at other points in the life cycle. Individual species phenological shifts in response to climatic variation are complex, and can include both environmental (cue‐driven) and organismal (response‐driven) mechanisms (Chmura et al., 2019). For example, monarch larval survival later in summer is lower than early season survival in the western United States, apparently due to changes in host plant quality and predator communities (Yang et al., 2020; Yang & Cenzer, 2020). This result broadly suggests that plant quality may vary seasonally. Later departure of monarchs from the central United States also could lead to mismatched phenology with nectar resources in the southern United States and northern Mexico (Inamine et al., 2016). Similarly, later departure could lead to a ‘developmental trap’ (Van Dyck et al., 2015), where insects initiate an additional late‐season generation with longer growing seasons, but do not have time to complete development before winter. In the case of monarchs, ‘completing development’ could refer to eggs that cannot develop into adults, or adults that emerge when conditions are no longer suitable for fall migration. Alternatively, a longer growing season can simply allow multivoltine insects to increase in abundance (Kerr et al., 2020). Clearly, understanding fall phenology of monarchs is important for conserving their North American migration, and the implications may vary among years based on weather conditions throughout the monarch's range.

Although overall monarch butterfly abundance did not change significantly through time, our monitoring data suggest that breeding season growth rates and peak migration abundance of monarchs at Camp Dodge may be declining through time. These patterns contradict a recent paper that used citizen science‐based counts to suggest that breeding‐season growth rates of monarchs have been increasing through time (Crossley et al., 2022). Citizen science‐based counts cover a large geographic area, but they are generally not as rigorously tabulated in comparison to a standardized single observer transect survey. A positive explanation of the trend we observed could be that newly restored habitat outside of Camp Dodge could potentially attract butterflies away from our transects, leading to an apparent decline. Alternatively, agricultural intensification in the landscape near Camp Dodge (Pleasants & Oberhauser, 2013) could reduce monarch growth rates on this landscape. A study of land conversion in this region found a significant net loss of grassland due to conversion of grassland to corn and soybean croplands from 2006 to 2011 (Wright & Wimberly, 2013). This loss of grassland in combination with the potential reduction in monarch breeding‐season population growth rates is concerning because more than half of monarchs overwintering in Mexico have natal origins in the central United States (Flockhart et al., 2017). The use of herbicides in agricultural landscapes can have negative implications for monarchs (Brower et al., 2006) and the effects of climate are generally becoming more apparent (Culbertson et al., 2022; Zylstra et al., 2021). Understanding the patterns and drivers of breeding‐season population growth rates will be a crucial complement to studies focused on multi‐year trends in monarch abundance.

The results of this research must be viewed within the context of the study's design, which was focused on one location within the central US landscape. Next steps should be to determine whether similar phenological patterns for both monarchs and milkweeds are being found across other locations in the region. In addition, conducting distance‐based sampling with a standard transect length could provide even better estimates of butterfly abundance, including detection probability. Finally, we only considered phenological changes for one host plant species (*A. syriaca*). Given other possible host plants (Pocius et al., 2017), it would be valuable to compare results with other milkweeds.

The original aim of our study was to investigate whether changes in early season phenology predict trends in abundance for monarch butterflies. The answer to this question is no, for the central United States. Currently, the start of breeding phenology is well‐matched to host plant availability and monarch larval success. These results, however, may not be generalizable across the entire monarch range. Shifts in early season phenology may have different implications for monarchs in hot, arid regions because there also may be issues with host‐plant biomass availability. Our results with respect to later fall departure of monarchs from their central US breeding ground deserve further attention. Causes and consequences should be examined, as it is unlikely that monarch phenology would shift independently of its local host plant and nectar resources. Nonetheless, our study contributes to a general pattern that negative impacts of phenological mismatches have been more elusive than some early studies indicated, as recently reviewed by Kharouba and Wolkovich (2023). Although our study raises new areas of concern for monarch butterfly conservation, it rules out at least one prominent possibility, specifically early season phenological mismatch (see also Gilmour & Kharouba, 2022) for monarch butterflies in the central United States.

## AUTHOR CONTRIBUTIONS

Diane M. Debinski, Norah Warchola, Sonia Altizer and Elizabeth E. Crone conceived the ideas and designed methodology; Norah Warchola collected the data; Elizabeth E. Crone and Diane M. Debinski analysed the data; Diane M. Debinski led the writing of the manuscript. All authors contributed critically to the drafts and gave final approval for publication.

## CONFLICT OF INTEREST STATEMENT

The authors declare no conflicts of interest.

## STATEMENT ON INCLUSION

Our study brings together four scientists from across the United States who have studied monarch butterflies from a variety of perspectives. All authors were engaged early on with the research and study design to ensure that the diverse sets of perspectives they represent was considered from the onset. One citizen scientist contributed valuable monarch monitoring data. Relevant literature published by scientists from across the globe was cited. Results of this research are being shared with local land managers, local and national conservation groups, as well as the scientific community.

## Supporting information


**Table S1.** AIC model comparison of cumulative growing degree days versus day of year as predictors of monarch butterfly sightings.
**Table S2.** Summary of numbers of larvae, pupae, and adults for each larval release group.
**Figure S1.** Visual inspection of phenology curves in relation to various metrics of temperature.

## Data Availability

The data that support the findings of this study are openly available in: https://github.com/elizabeth‐crone/Camp‐Dodge‐Monarchs.
