# Supplementary material for: Implications of summer breeding phenology on demography of monarch butterflies
Source: J Anim Ecol. 2025 Feb 17;94(4):682–92. doi: 10.1111/1365-2656.70004 (PMC11962244; doi:10.1111/1365-2656.70004)
Supplement: Supplementary file 1 — Table S1. AIC model comparison of cumulative growing degree days versus day of year as predictors of monarch butterfly sightings. Table S2. Summary of numbers of larvae, pupae, and adults for each larval release group. Figure S1. Visual inspection of phenology curves in relation to various metrics of temperature. [file JANE-94-682-s001.docx]

Supplemental Table 1. AIC model comparison of Cumulative Growing Degree Days vs. Day of Year as predictors of monarch butterfly sightings. DOY= day of year, cGDD = cumulative growing degree days.

dAIC df

DOY*year 0.0 18.9

DOY 5.4 9.3

DOY+year 7.3 10.3

cGDD 51.2 9.9

cGDD*year 51.6 16.0

cGDD+year 52.9 10.9

Notes: Growing degree days (GDD) is a time-based measure of heat accumulation, calculated by accumulating the daily total of degrees above a temperature threshold (Cayton et al., 2015). GDD has a long history of application in agriculture and has more recently been adopted by ecologists to link climate change to phenological shifts (Cayton et al., 2015; Diamond et al., 2014). GDD data were downloaded from Iowa State University’s Iowa Environmental Mesonet <https://mesonet.agron.iastate.edu/plotting/auto/?q=108> and calculated using a baseline of 4.44 ⁰C (default setting) and a maximum of 30 ⁰C from the weather station located in Knoxville, Iowa. Cumulative Growing Degree Days is the sum of the GDD from the start of the time of heat accumulation to the date of interest.

Supplemental Table 2. Summary of numbers of larvae, pupae, and adults for each larval release group.

| Release group | # larvae | Est # pupae^1^ | # adults |
| --- | --- | --- | --- |
| Earlier | 420 | 10 | 5 |
| Current | 420 | 100 | 51 |
| Later | 280 | 48 | 19 |
| ^1^Not uniquely marked, so counts are uncertain | | | |

Supplemental Figure 1. Visual inspection of phenology curves in relation to various metrics of temperature: (A) total cumulative growing degree days (cGDD) in each year, (B) average daily GDD in each year, (C) slope of cumulative GDD vs. day of year (DOY) in each year (see Fig 2C. In main text). Colors range from coolest (yellow) to warmest (red) by each ranking. In no case is there a clear pattern of phenology with temperature. These results complement the overall AIC model fitting result that day of year was a better predictor of abundance and phenology than GDD (see Supplemental Table 1).

| 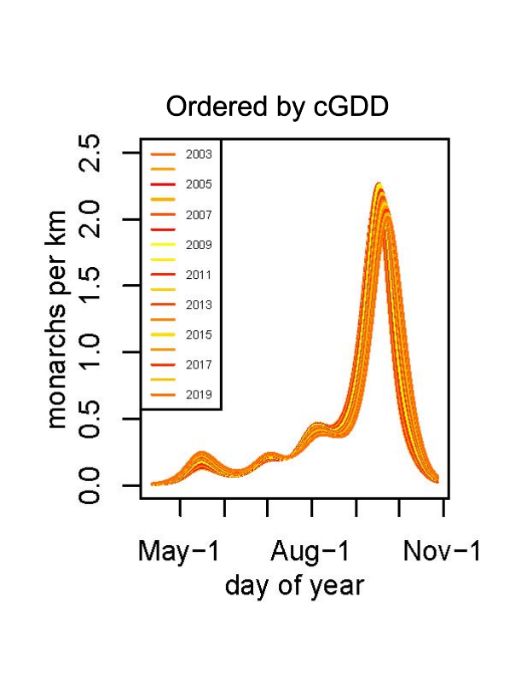 | 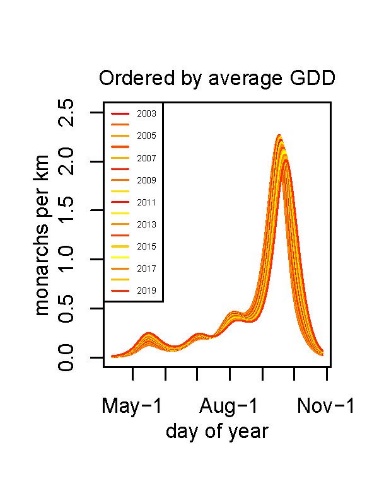 | 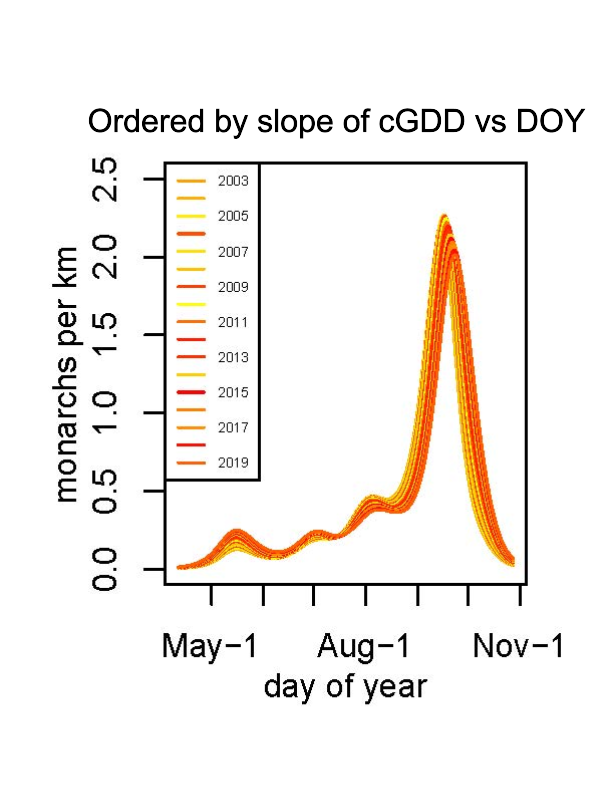 |
| --- | --- | --- |
